# Transcriptome analysis to identify candidate genes related to mammary gland development of Bactrian camel (*Camelus bactrianus*)

**DOI:** 10.3389/fvets.2023.1196950

**Published:** 2023-06-05

**Authors:** Huaibing Yao, Xiaorui Liang, Zhihua Dou, Zhongkai Zhao, Wanpeng Ma, Zelin Hao, Hui Yan, Yuzhuo Wang, Zhuangyuan Wu, Gangliang Chen, Jie Yang

**Affiliations:** ^1^Key Laboratory of Biological Resources and Genetic Engineering, College of Life Science and Technology, Xinjiang University, Ürümqi, China; ^2^Xinjiang Camel Industry Engineering Technology Research Center, Ürümqi, China; ^3^College of Veterinary Medicine, Xinjiang Agricultural University, Ürümqi, China; ^4^Xinjiang Altai Regional Animal Husbandry Veterinary Station, Altay, China; ^5^Bactrian Camel Academy of Xinjiang, Wangyuan Camel Milk Limited Company, Altay, China

**Keywords:** Bactrian camel, transcriptome sequencing, mammary gland, development, differentially expressed gene

## Abstract

**Introduction:**

The demand for camel milk, which has unique therapeutic properties, is increasing. The mammary gland is the organ in mammals responsible for the production and quality of milk. However, few studies have investigated the genes or pathways related to mammary gland growth and development in Bactrian camels. This study aimed to compare the morphological changes in mammary gland tissue and transcriptome expression profiles between young and adult female Bactrian camels and to explore the potential candidate genes and signaling pathways related to mammary gland development.

**Methods:**

Three 2  years-old female camels and three 5  years-old adult female camels were maintained in the same environment. The parenchyma of the mammary gland tissue was sampled from the camels using percutaneous needle biopsy. Morphological changes were observed using hematoxylin-eosin staining. High-throughput RNA sequencing was performed using the Illumina HiSeq platform to analyze changes in the transcriptome between young and adult camels. Functional enrichment, pathway enrichment, and protein–protein interaction networks were also analyzed. Gene expression was verified using quantitative real-time polymerase chain reaction (qRT-PCR).

**Results:**

Histomorphological analysis showed that the mammary ducts and mammary epithelial cells in adult female camels were greatly developed and differentiated from those in young camels. Transcriptome analysis showed that 2,851 differentially expressed genes were obtained in the adult camel group compared to the young camel group, of which 1,420 were upregulated, 1,431 were downregulated, and 2,419 encoded proteins. Functional enrichment analysis revealed that the upregulated genes were significantly enriched for 24 pathways, including the Hedgehog signaling pathway which is closely related to mammary gland development. The downregulated genes were significantly enriched for seven pathways, among these the Wnt signaling pathway was significantly related to mammary gland development. The protein–protein interaction network sorted the nodes according to the degree of gene interaction and identified nine candidate genes: *PRKAB2*, *PRKAG3*, *PLCB4*, *BTRC*, *GLI1*, *WIF1*, *DKK2*, *FZD3*, and *WNT4*. The expression of fifteen genes randomly detected by qRT-PCR showed results consistent with those of the transcriptome analysis.

**Discussion:**

Preliminary findings indicate that the Hedgehog, Wnt, oxytocin, insulin, and steroid biosynthesis signaling pathways have important effects on mammary gland development in dairy camels. Given the importance of these pathways and the interconnections of the involved genes, the genes in these pathways should be considered potential candidate genes. This study provides a theoretical basis for elucidating the molecular mechanisms associated with mammary gland development and milk production in Bactrian camels.

## 1. Introduction

Bactrian camels (two-humped camels), are mammals of the genus Camelidae of the family Mammalia that mainly inhabit cooler areas in Asia (1). There are five local species of Bactrian camels in China, namely Junggar, Tarim, Alashan, Sunit, and Qinghai, which are distributed in the arid and semi-arid regions of the Xinjiang, Inner Mongolia, Gansu, and Qinghai provinces in northwestern China. The Bactrian camel grazes a wide range of desert plants, can tolerate extreme environments, and has a specialized immune system. It is a distinctly important economic animal resource in desert areas, providing livestock products such as milk, meat, hides, and wool to herders in remote areas. In addition, camel milk has a high nutritional value, is hypoallergenic, and contains small-molecule nano-antibodies that can assist in the treatment of multifunctional disorders and autoimmune diseases such as lung cancer and diabetes (2, 3). However, camels grow slowly, reaching puberty at a later age than other livestock species. Sexual maturity is probably reached at 3–4 years. Female Bactrian camels give birth to their first calves at the age of 4 or 5 and produce offspring once every two years with a gestation period of 13–14 months. This slow reproductive rate, combined with the small geographical distribution, free-range grazing, and traditional breeding, results in low milk production and a shortage of camel milk supply to the market. Camels are crucial to the economies of many countries in arid and semi-arid regions of the world and camel breeding is thus increasing annually worldwide. With this increase, the metabolic processes and molecular mechanisms related to adaptation to extreme desert environments have been identified by genome sequencing and comparative genome analysis of Bactrian camels and dromedaries (4). There is a crucial need for developing knowledge of the genetic and molecular mechanisms related to milk production traits in Bactrian camels. Elucidating the mechanisms of mammary gland development is an initial key step to achieve this.

The mammary gland develops and produces milk under the regulation of systemic hormones, which is a dynamic and highly complex multistep process involving the periodic cycling of mammary epithelial proliferation, differentiation, and apoptosis (5). The components of milk are derived from blood-circulating nutrients, and they are synthesized in the epithelial cells of the mammary gland in lactating animals. Almost all studies on milk production traits have been conducted based on analysis of mammary glands and animal hematology (6). Mammary tissues undergo rapid growth during adolescence and develop again during pregnancy until lactation is complete (7). Animal studies have revealed apparent anatomical and physiological differences between juvenile and adult mammary glands. Therefore, the development and function of the mammary glands are essential for the provision of nutrition to offspring and the study of milk production traits. However, there are limitations in the analysis of milk performance traits using mammary tissues, such as difficulties in sampling, damage to animas, and ethical considerations.

Functional genomic tools have been widely used to study the molecular mechanisms of growth, development, and production traits in livestock (8, 9). Transcriptome analysis has been used to identify the molecular information that regulates economically important traits of an organism. Recently, researchers have studied the molecular mechanisms underlying mammary gland development in various species, such as cattle (10, 11), sheep (12), cats (13), rabbits (13), and rats (14). However, to date, the mechanisms underlying mammary gland development and the initiation of lactation have not been reported in Bactrian camels. The effects of specific candidate genes on physiology, mammary gland development and milk production traits remain unknown. Therefore, the current study uses transcriptome analysis of the mammary gland tissues of young and adult camels to investigate important candidate genes and pathways involved in mammary gland development in Bactrian camels.

## 2. Materials and methods

### 2.1. Experimental camels and sample collection

The transcriptome sequencing sample population consisted of 6 domestic Junggar Bactrian camels with no consanguineous relationships among them, including adolescent young camels (YTR; *n* = 3; 2 years old) and lactating adult female camels (CNR; *n* = 3; 5 years old) from Fuhai County, Altay Prefecture, northwest China’s Xinjiang Uygur Autonomous Region (87° 35′25′′E, 46° 86′6′′N). The mammary glands of 2 years-old adolescent female camels are in the developmental stage and do not lactate. At 4 or 5 years of age, female camels begin to sexually mature until the end of their first pregnancy, when their mammary glands are fully developed and they have the ability to secrete milk after giving birth. All experimental camels were fed under the same conditions to reduce the environmental effects on gene expression.

**Figure 1:**
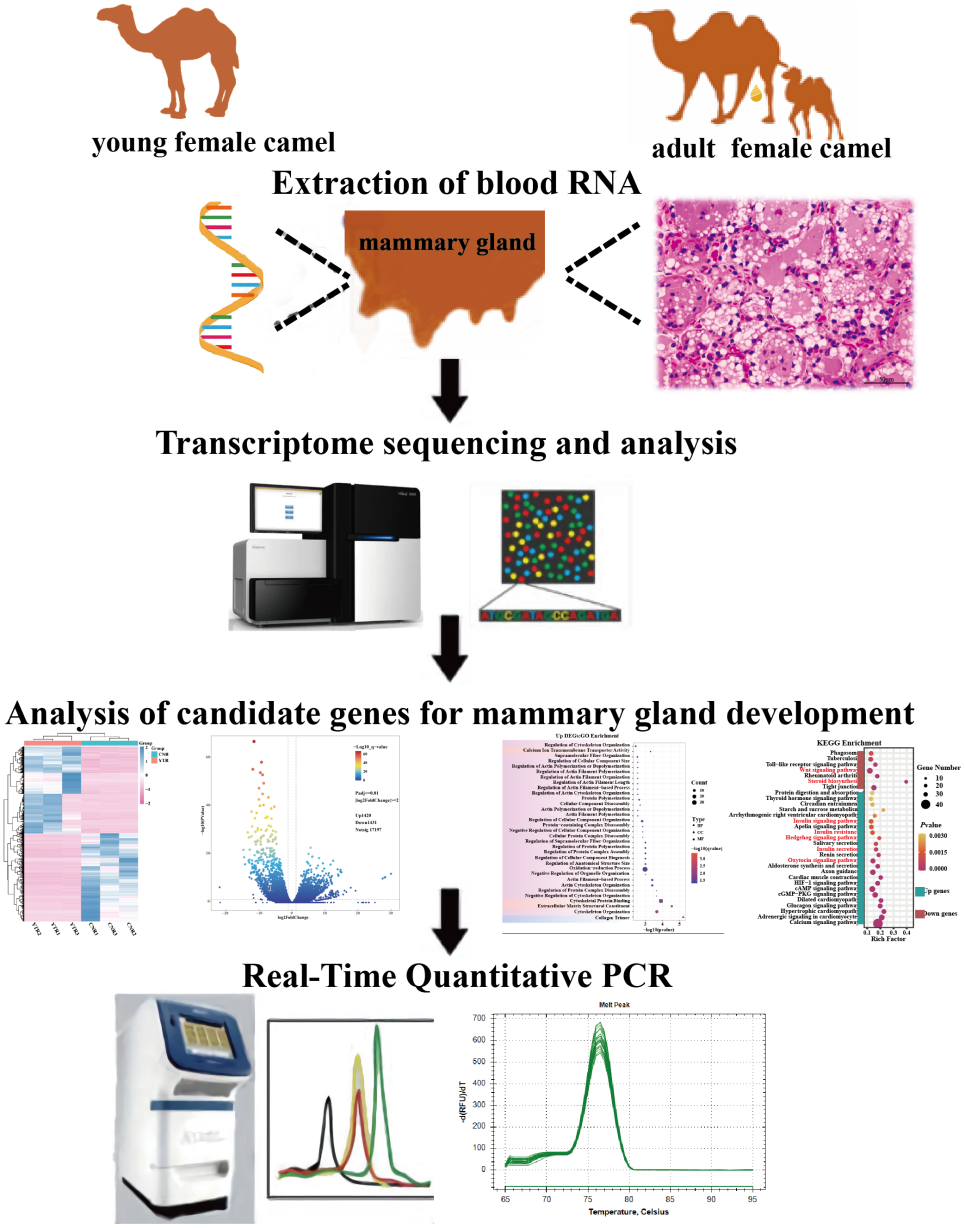
Experimental design of the study.

A specialist veterinarian conducted sampling to minimize harm to the camels. The parenchyma of the mammary gland tissue, which contains lobules that synthesize milk (15), was harvested from camels by percutaneous needle biopsy. Part of the tissue samples were immediately frozen in liquid nitrogen then transferred to the laboratory and stored at −80°C for subsequent RNA extraction. The remaining mammary gland tissues were washed with sterile saline and preserved in a 2.5% glutaraldehyde solution for later use in hematoxylin-eosin (H&E) staining (Figure 1). All the experimental procedures were performed in accordance with the guidelines of the Laboratory Animal Administration Regulations issued by the National Science and Technology Committee (China). All experimental animal studies were reviewed and approved by the Animal Welfare Committee of Xinjiang University (approval ID: XJU2019012).

### 2.2. Micromorphological examination

The mammary gland tissues obtained from the experimental camels were fixed in 10% neutral buffered formalin, dehydrated in a graded alcohol series, cleared in xylol, embedded in paraffin, and sectioned to a thickness of 4–5 μm. Tissue sections were prepared and stained with H&E following the protocols of a previous study (16) and photographed under a light microscope (Eclipse E100 Nikon, Japan).

### 2.3. RNA extraction, quality, and integrity determination

Total RNA was extracted from the camel mammary tissue using a TRIZOL RNA Extraction Kit (Invitrogen, Carlsbad, CA, United States). RNA integrity was evaluated using agarose 1.0% gel electrophoresis and the RNA Nano 6,000 Assay Kit of the Agilent 2,100 Bioanalyzer (Agilent, Santa Clara, CA, United States). RNA samples that passed quality control were used for subsequent sequencing library construction.

### 2.4. Library construction and transcriptome sequencing

Library preparation and transcriptome sequencing were performed by Novogene Co., Ltd. (Tianjin, China). The mRNA was purified from 1 μg of total RNA using oligo dT magnetic beads. mRNA was fragmented using divalent cations at high temperatures. After fragmentation, the cDNA library was synthesized using random hexamer primers, M-MuLV Reverse Transcriptase, and paired-end sequencing using the HiSeq 6000 platform (Illumina) (17). Reads containing adapters, poly-N, and low-quality reads were removed to obtain clean reads by Fastp (v 0.19.7). The Q20, Q30, and GC contents of the clean data were calculated and all downstream analyses were performed using high-quality clean data. A set of genomic index files of *Camelus bactrianus* reference genome was built using Hisat2 (version 2.0.5) and clean paired-end reads were mapped to the reference genome using Hisat2. Gene expression levels were quantified using featureCounts (version 1.5.0-p3) and the fragments per kilobase of transcript per million mapped reads (FPKM) of each annotated gene were counted based on the length of the gene and the read counts mapped to this gene (18).

### 2.5. Differentially expressed genes identification

Principal component analysis (PCA) was used to visually identify differences between the different sequencing sample groups in the transcriptome data. Differential expression analysis was performed between the two groups of camels using the DESeq2 package in R (version 4.2.1), based on negative binomial distribution. Considering previous studies (19, 20) and our specific experimental situation, adjusted *p*-values (*p*_adjust_) < 0.01 and |log2FoldChange| >2 were established as the thresholds for significance. *p*-values were adjusted for multiple testing using the Benjamini and Hochberg methods. Genes with *p*_adjust_ < 0.01 and log2FoldChange >2 by DESeq2 were defined as upregulated differentially expressed genes (DEGs); genes with *p*_adjust_ < 0.01 and log2FoldChange <−2 were regarded as downregulated DEGs.

### 2.6. Gene functional enrichment analysis of DEGs

To explore functions and pathways associated with the DEGs, Gene Ontology (GO) and Kyoto Encyclopedia of Genes and Genomes (KEGG) pathway enrichment analyses were performed using the NovoMagic Cloud Platform.^1^ A cut-off of *p* < 0.05 was used to screen significant functions and pathways. We further screened the core pathways and candidate genes by systematically reviewing the literature.

### 2.7. Protein–protein interaction network of DEGs

Gene regulatory networks have elucidated the regulation of mammary gland development in animals (21, 22). Therefore, we uploaded all identified DEGs to the Search Tool for the Retrieval of Interacting Genes/Proteins database^2^ to create a protein–protein interaction (PPI) network (23). PPI networks were established using Cytoscape software (version 4.8.0). Hub genes were identified and screened by network analysis using Cytoscape and its plugin (CytoHubba) (24).

### 2.8. Quantitative real-time polymerase chain reaction validation of DEGs

The Prime Script RT Reagent Kit (Takara Biotechnology Co., Ltd., Shiga, Japan) was used to produce cDNA from RNA. The obtained cDNA was analyzed immediately or stored at −20°C. Camel beta-actin (β-actin) (NCBI accession no. XM_010965866.2) was used as an internal reference gene (25). Fifteen genes were selected from the DEGs between the young camel and adult camel groups at another sampling site. Polymerase chain reaction (PCR) was performed using the following cycling conditions: 95°C for 5 min; 40 cycles of 95°C for 10 s and 60°C for 30 s; 95°C for 15 s, 60°C for 60 s, and 95°C for 15 s. Specific primers were designed using the Primer-BLAST online website. Three replicates of each sample were used to calculate the relative expression by the 2^−ΔΔct^ method (26). The identities of these DEGs and specific primer sequences are listed in Table 1.

**Table 1:** The primers used for RT-qPCR are listed (F = forward, R = reverse).

Gene	Primer sequence (5′ → 3′)	Annealing temperature (°C)	Product length (bp)
*β-actin*	F: CAGATCATGTTCGAGACCTTC	55	275
	R: ATGTCACGCACGATTTCC		
*CAMK2D*	F: CTCTCCTGTAGGAAGCAACCAG	56	101
	R: CCTTTGCCATCCATCCCACT		
*CAMK2A*	F: CGATGACTCTCCTTTTCTCC	54	105
	R: CTCCCAAGTTTCTTCTTGGA		
*BTRC*	F: GAGAGATACTATGGCTACACTG	55	114
	R: GCTCAGTGTTCTTTCCATCAG		
*PRKAB2*	F: GGATCCCGATGGAGAAGT	55	104
	R: GGAAGTCTGAAGTAGAAGCC		
*PLCB4*	F: CTGCTCACTCAAAAGGATCT	55	104
	R: GTGAGTGAAGTTTCTGGGTA		
*PPP1R3A*	F: TTAGGCACAGAAAGGTGAAG	56	102
	R: GCTCGTTTCACAGTTGAGTA		
*SLC1A1*	F: TACGCTTATTTCTGGGTGAG	54	102
	R: AACCTCCTTCTTCTCTTCCT		
*LTF*	F: ACCAAGGAAGAATCATCACC	55	111
	R: CCGGGTATTTGTTGTTAAGC		
*PIGR*	F: AGTTGCTCTCACCAATAAGG	55	109
	R: GTTCACTCTGCTTAGCTCTT		
*GLI1*	F: CAGAGAGACCAACAGCTGCA	54	112
	R: CAGGCTGGCATCCGATAGAG		
*WIF1*	F: CAGGCGAGAGTGCTCATAGG	56	118
	R: TGACAGGAATCGCTGGCATT		
*DKK2*	F: TCGGCACAGAGATCGAAACC	54	171
	R: TTGGTCCAGAAGTGACGAGC		
*FZD3*	F: CGATCGAGGAAGCATGGCTA	56	126
	R: TCTTGGCACATCCTCAAGGT		
*WNT4*	F: TATCCTGACACACATGCGGG	56	139
	R: CTCGGTGGCTCCATCAAACT		
*KCNJ12*	F: CATCACCATCCTGCACGAGA	54	160
	R: CTCATTGGCCAGGTAGGAGC		

## 3. Results

### 3.1. Histomorphometric analysis of mammary gland

**Figure 2:**
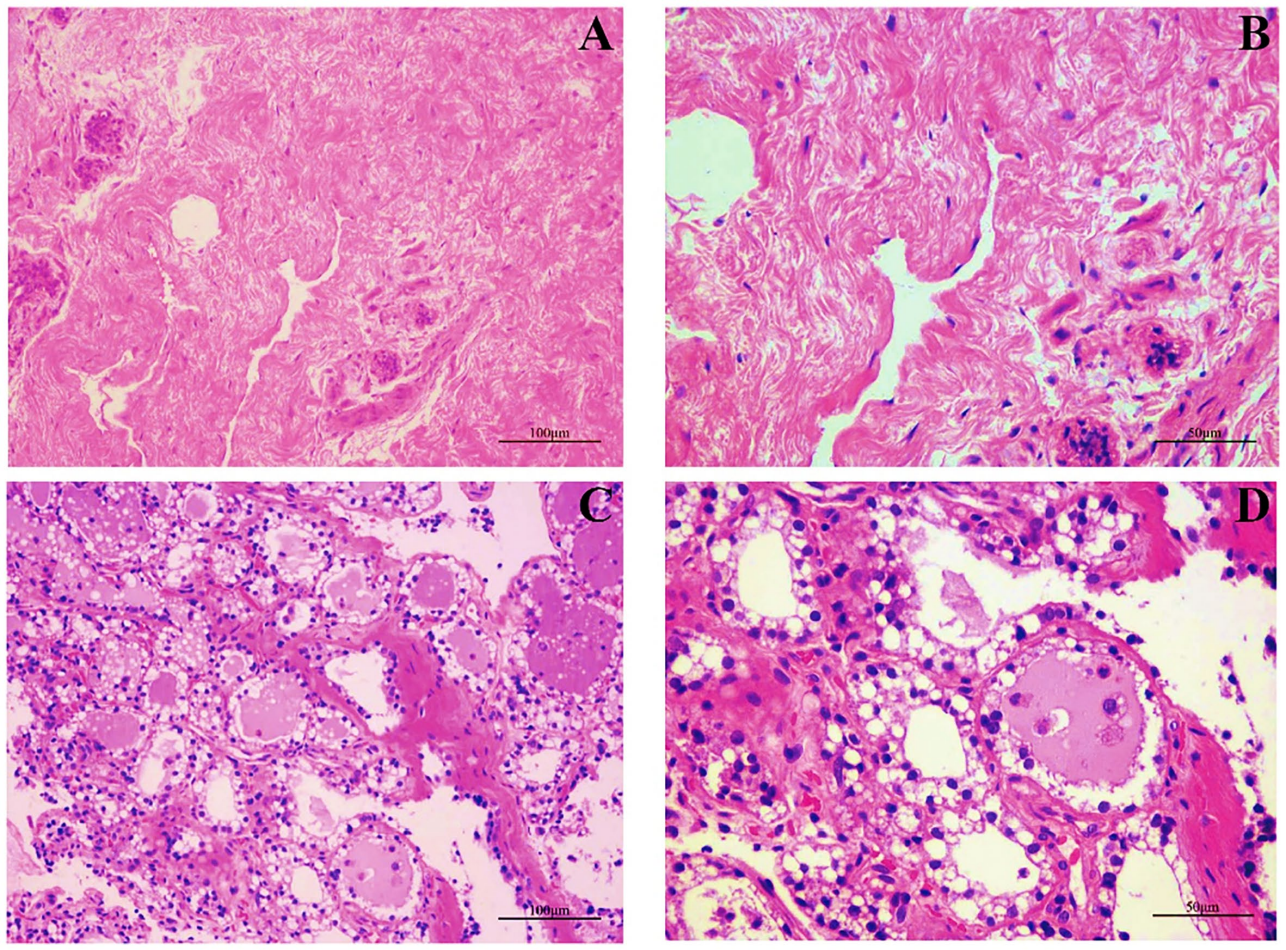
Mammary gland morphology in young and adult female camels. **(A)** Morphological structure of the mammary glands of young camels (200×). **(B)** Morphological structure of the mammary glands of young camels (400×). **(C)** Morphological structure of the mammary glands of adult camels (200×). **(D)** Morphological structure of the mammary glands of adult camels (400×).

We first examined the morphological and microscopic characteristics of camel mammary glands according to published literature on other species. The morphological differences at different stages of mammary gland development are shown in Figure 2. As reported for other young animals (27–29), the mammary gland tissue of young camels was incompletely developed and consisted of a large amount of connective tissue (Figures 2A,B). Furthermore, the terminal duct lobular units (TDLU) in the mammary gland were incompletely developed, with inner epithelial tissue surrounded by lobular connective tissue and an outer overlay of interlobular connective tissue, and with fewer and irregularly shaped ducts and epithelial cells. The mammary glands of adult female camels were more fully developed (Figures 2C,D), with a large number of mammary epithelial cells and uniform chromatin distribution, adipose tissue, a small amount of thin connective tissue, and fat pads scattered with alveoli, ducts, and alveolar cavities.

### 3.2. RNA-sequencing reads and mapping to the reference genome

Raw reads were obtained for each library and between 43 and 59 million clean reads were obtained after quality control procedures and applied to the YTR and CNR libraries (Table 2). The Q30 reads (quality score >97.69%) were >95% for all samples (Supplementary Table S1). Between 38 and 53 million reads were mapped to the Bactrian camel genome for the YTR and CNR groups, respectively. Approximately 92% of the clean reads mapped to the camel reference genome, of which nearly 89% were uniquely mapped. The detailed statistical information is provided in Supplementary Table S2. These data provided a solid basis for subsequent analyses.

**Table 2:** Summary of the RNA-seq analyses after mapping to the reference genome.

Sample	Raw reads	Clean reads	GC content	Total map	Unique map	Multi map
YTR1	59,165,478	58,350,742	51.57	53,783,775 (92.17%)	52,472,376 (89.93%)	1,311,399 (2.25%)
YTR2	46,255,466	45,642,826	51.48	42,138,839 (92.32%)	41,094,703 (90.04%)	1,044,136 (2.29%)
YTR3	43,870,868	41,787,324	48.54	38,482,571 (92.09%)	37,891,330 (90.68%)	591,241 (1.41%)
CNR1	46,672,290	45,317,128	42.60	42,437,154 (93.64%)	41,233,336 (90.99%)	1,203,818 (2.66%)
CNR2	47,309,962	46,649,412	47.39	44,449,779 (95.28%)	42,274,957 (90.62%)	2,174,822 (4.66%)
CNR3	49,864,878	49,116,410	45.86	47,175,819 (96.05%)	44,318,893 (90.23%)	2,856,926 (5.82%)

### 3.3. Analysis of DEGs

**Figure 3:**
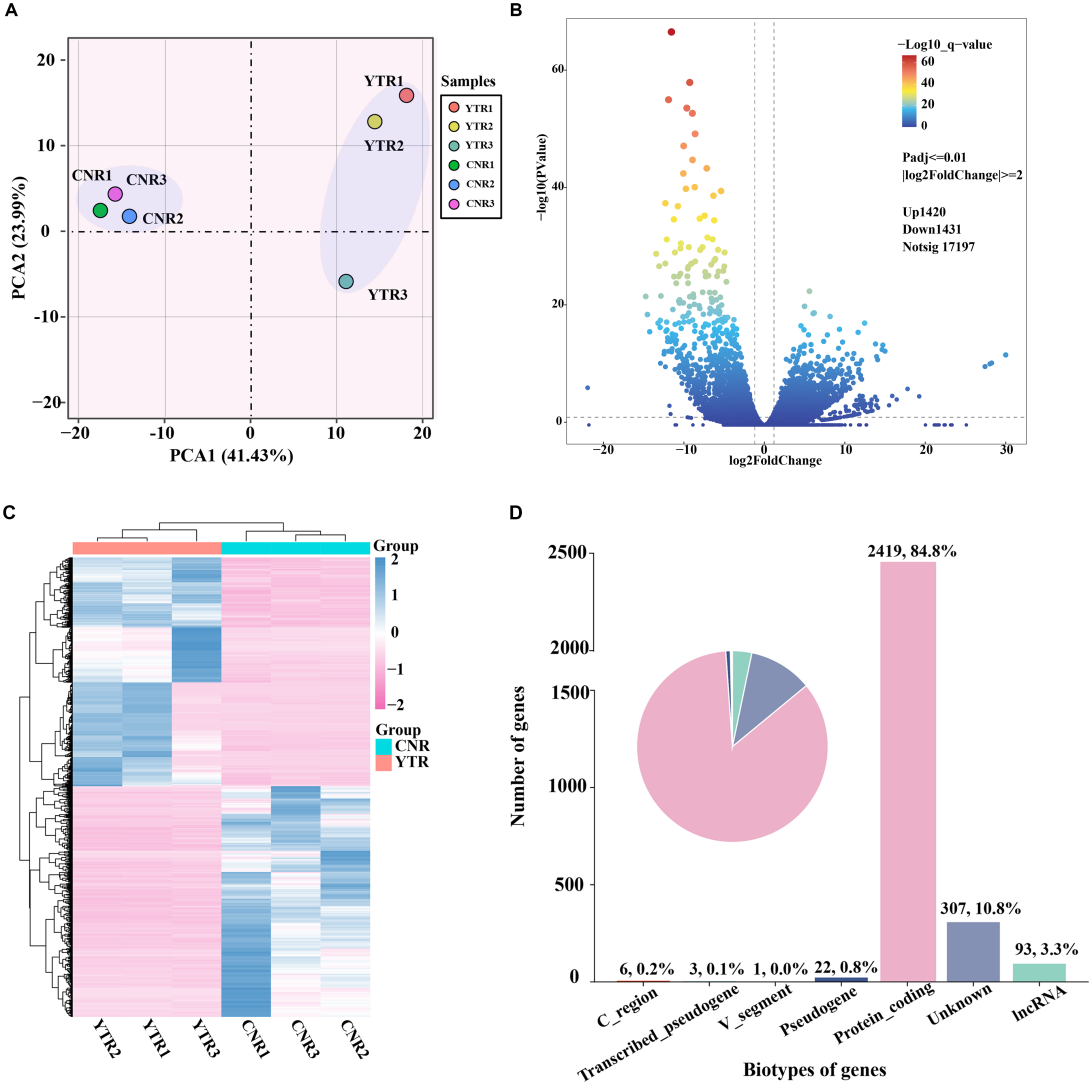
Analysis of differentially expressed genes among sample groups. **(A)** Principal-component analysis comparing the two sample groups. **(B)** Volcano plot of differentially expressed genes. **(C)** Heatmap and the hierarchical cluster analysis of the differentially expressed genes. **(D)** Biotype of genes statistical analysis.

The number of genes at different expression intervals was determined (Supplementary Table S3). The first principal component (PCA1), which had the largest variance (41.43%), distinctly clustered the samples into two groups, with three biological replicates within each group clustered together (Figure 3A). A total of 2,851 genes were identified as differentially expressed: 1,420 (49.81%) were upregulated and 1,431 (50.19%) were downregulated (YTR vs. CNR) (Figure 3B and Supplementary Table S4). The expressed genes were subjected to hierarchical clustering to comprehensively and intuitively display the differences in gene expression between the two groups (Figure 3C). Statistical analysis of the gene biotypes was performed on the DEGs to identify the composition of gene categories. Of these, 2,419 (84.8%) were protein-coding genes, followed by unknown type genes (10.8%) and 93 (3.3%) long non-coding RNA genes (Figure 3D).

### 3.4. Go enrichment analysis

**Figure 4:**
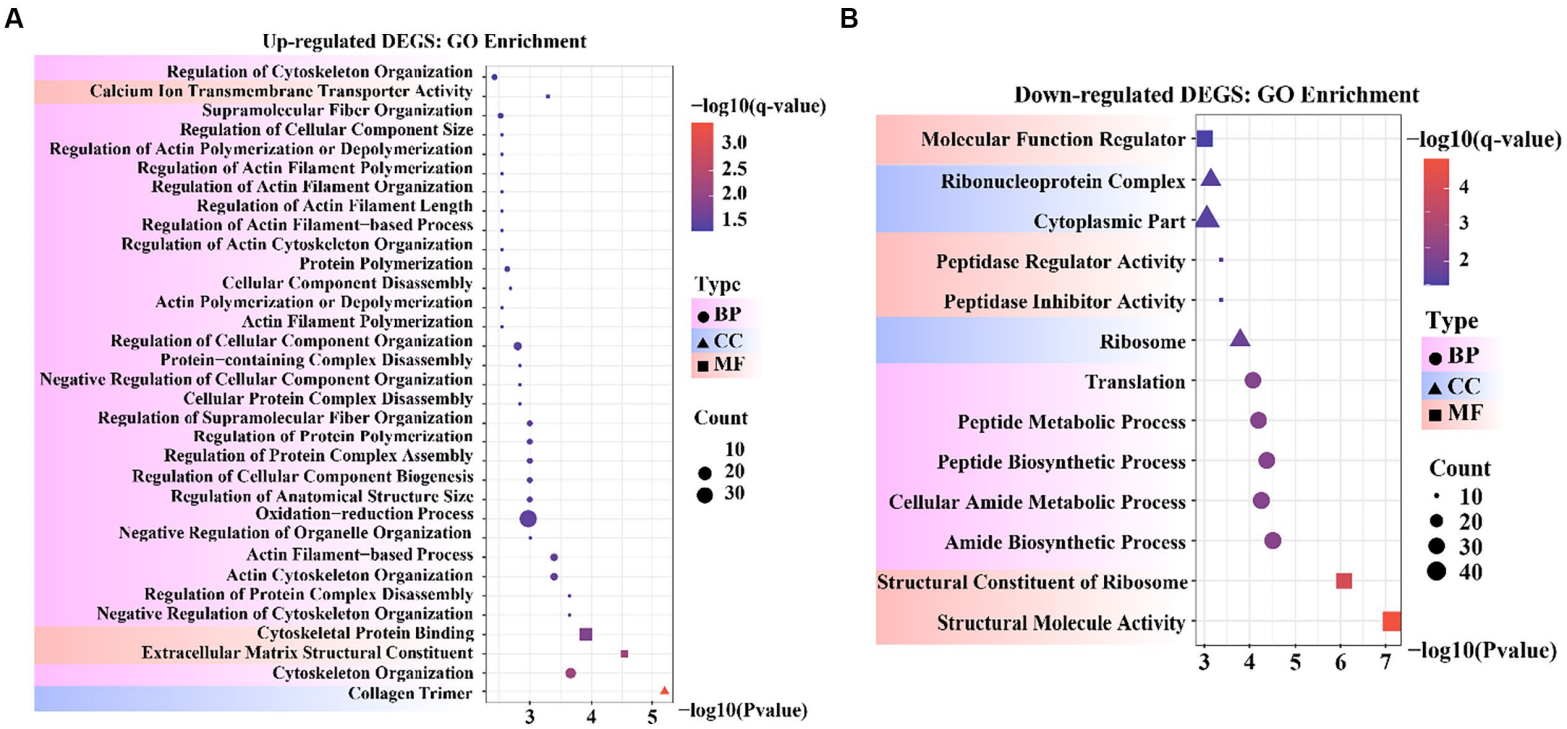
Bubble diagram of Gene Ontology (GO) enrichment result. **(A)** GO enrichment data for genes that were up-regulated. **(B)** GO enrichment data for genes that were down-regulated.

To obtain a more comprehensive understanding of the DEGs, we performed GO functional enrichment analysis using DAVID. These DEGs were enriched in 827 GO terms, of which 434 were related to biological processes (BP), 279 to molecular functions (MF), and 114 to cellular components (CC). Moreover, collagen trimers and extracellular matrix structural constituents were significantly enriched (*p*_adjust_ < 0.05). The complete GO analysis results are provided in Supplementary Table S5. Significance analysis of GO enrichment of upregulated DEGs revealed 33 significantly enriched entries (*p*_adjust_ < 0.05), including 29 BP terms, 3 MF terms, and 1 CC term (Supplementary Table S6). These terms were related to cytoskeletal organization, organelle regulation, actin regulation, protein organization, cytoskeletal and component organization regulation, and calcium ion transport across membranes (Figure 4A).

GO enrichment of the downregulated DEGs revealed 13 significantly enriched entries (*p*_adjust_ < 0.05), including five BP terms, five MF terms, and three CC terms (Supplementary Table S7). These terms were involved in molecular functional regulators, riboprotein complexes, cytoplasmic fraction, peptidase regulator activity and inhibitor activity, ribosomes, translation, peptide metabolism and peptide biosynthesis processes, cellular amide metabolism processes, amide biosynthesis processes, ribosome structural components, and structural molecular activity (Figure 4B). In the reference GO terms reported in previous studies, these significantly enriched GO entries were intimately correlated with the maintenance of cell morphology and structure, organismal tissue growth and development, and response to cell growth. Among these, peptidase regulator activity and inhibitor activity entries can regulate aminopeptidase N to promote mammary gland development in animals.

### 3.5. KEGG pathway enrichment analysis

Signaling pathways are essential for mammary gland development. We performed KEGG pathway enrichment analysis to investigate the gene-enriched pathways. Overall, 2,851 DEGs were assigned to 318 KEGG pathways, of which 11 were significantly enriched (*p*_adjust_ < 0.05). The main pathways were insulin secretion, calcium and glucagon signaling pathway, steroid biosynthesis, HIF-1 signaling pathway, protein digestion and absorption, adrenergic signaling in cardiomyocytes, and tight junction (Supplementary Table S8).

**Figure 5:**
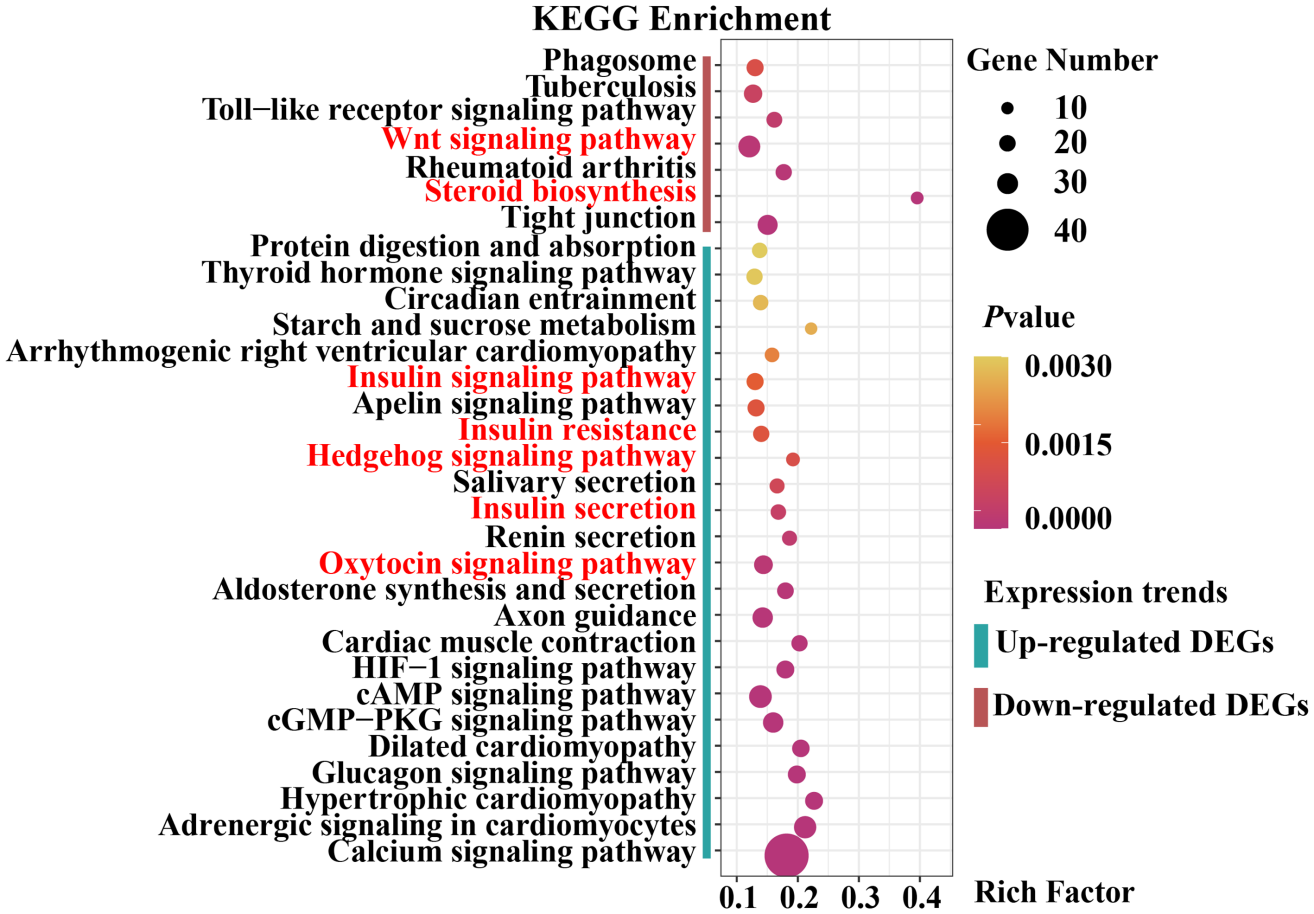
Dot plot of Kyoto Encyclopedia of Genes and Genomes (KEGG) pathway enrichment analysis results.

The upregulated and downregulated genes were subjected to KEGG pathway enrichment analysis. The enrichment analysis results indicated that 24 pathways were significantly up-regulated and 7 were significantly down-regulated (*p*_adjust_ < 0.05) (Figure 5). Upregulated genes were significantly enriched in the Hedgehog, insulin, and oxytocin signaling pathways (Supplementary Table S9). Downregulated genes were significantly enriched in the Wingless-Type MMTV Integration Site Family (Wnt) signaling pathway, steroid biosynthesis, and tight junctions (Supplementary Table S10). As previously reported, the Hedgehog signaling pathway (30, 31), Wnt signaling pathway (32, 33), hormone-related pathways (34–36), and tight junctions (37) are closely associated with mammary gland development in animals. Collectively, these results provide a basis for selecting candidate genes related to mammary gland growth and development.

### 3.6. PPI network of the DEGs

**Figure 6:**
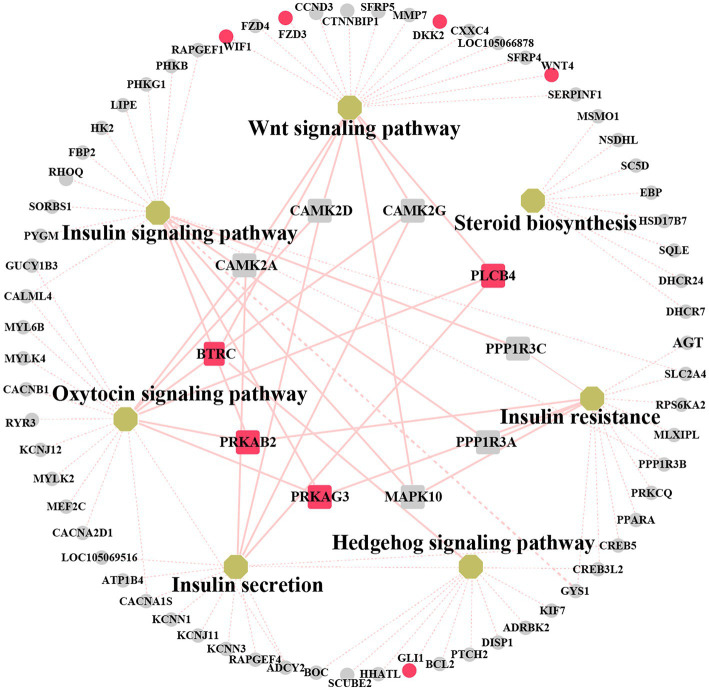
Networks analysis between protein–protein interaction (PPI) network and KEGG pathway related to mammary gland development. Non-hub genes are represented as grey circle while yellow octagons represent pathways. Hub genes are indicated by rectangles. The important candiate genes are highlighted in red. The light red solid line indicates the high degree of gene linkage. The light red dashed line indicates the low level of gene connectivity.

We explored the biological and regulatory functions of hub DEGs at the protein level. Next, we collated the genes in important pathways for the (PPI) network analysis and sorted the nodes according to the degree of interaction (Figure 6 and Supplementary Table S11). Among the top 10 central genes, *PRKAB2* and *PRKAG3* are associated with animal growth, slaughter, and meat quality traits (38); *PLCB4* genes are important candidate genes for pig growth and development and average daily weight gain (39). *BTRC* affects spermatogenesis and mammary gland development in mice (40). The four hub genes showed a high degree of interaction. Notably, some non-hub genes, such as *GLI1*, *WIF1*, *DKK2*, *FZD3*, and *WNT4* genes, have been reported to be associated with mammary gland development. This demonstrated that these genes play important roles in animal growth and development.

### 3.7. Validation of gene expression

**Figure 7:**
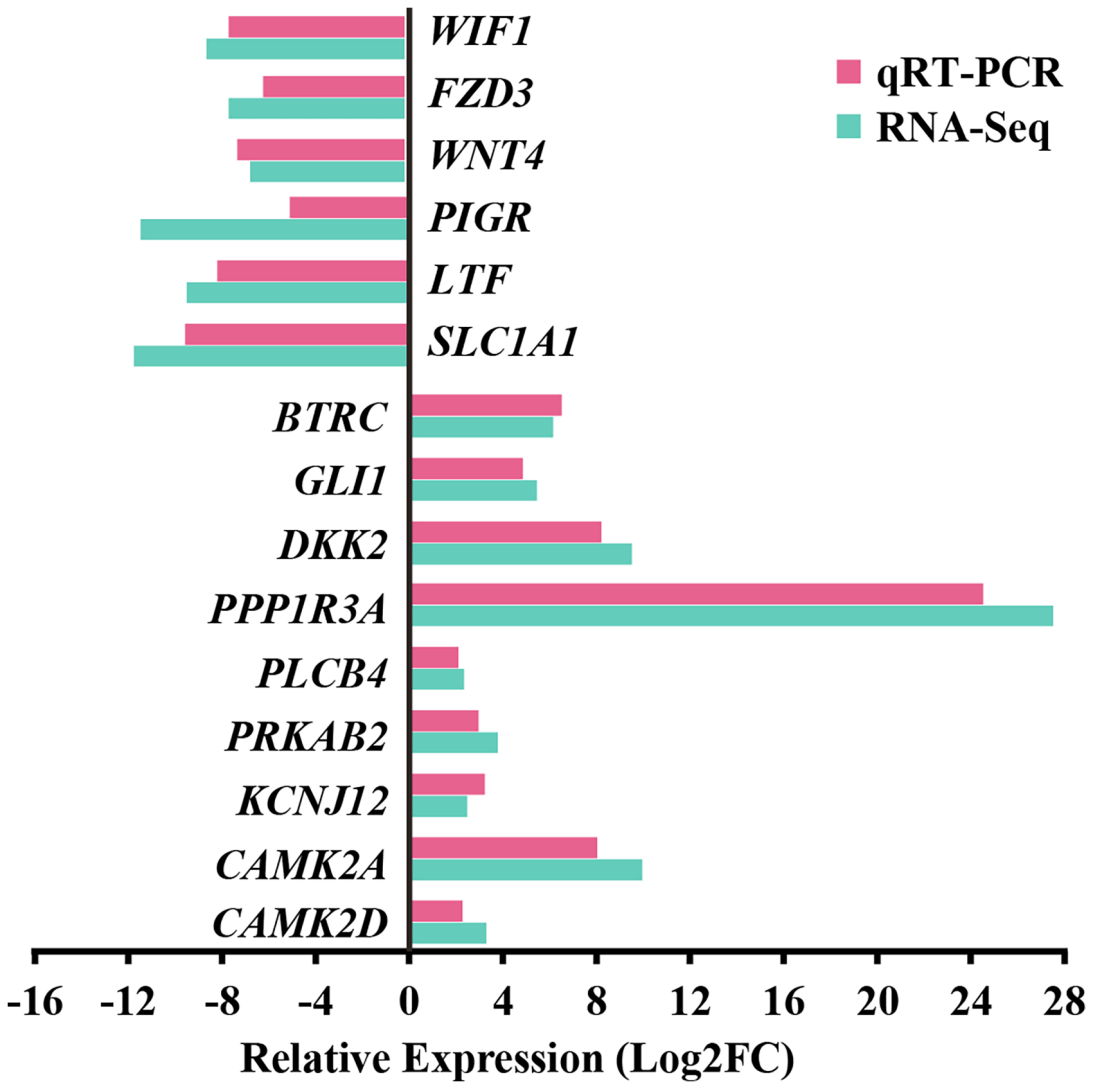
Validation of RNA-seq differential gene expression levels using RT-qPCR. The relative expression levels of both the RT-qPCR and RNA-seq were expressed as log2FoldChange (FC), which is defined as the ratio of the expression level in the young camels to that in the adult camels for a specific gene. Error bars are standard deviations across three camels per group evaluated.

For the preliminary validation of the RNA-sequencing data, fifteen DEGs were selected for experimental validation and statistical analysis in another random camel population, including nine upregulated genes (*CAMK2D, CAMK2A, BTRC, PRKAB2, PLCB4, PPP1R3A, GLI1, DKK2*, and *KCNJ12*) and six downregulated genes (*SLC1A1, LTF, WIF1, FZD3, WNT4*, and *PIGR*). The gene expression levels were normalized relative to β-actin. The expression trends of fifteen genes in the random camel population were consistent with the transcriptome results (Figure 7 and Supplementary Table S12).

## 4. Discussion

Camels, like cattle, have four mammary glands in the inguinal region responsible for the synthesis and secretion of milk proteins (41). Because camels do not have gland cisterns for storage, milk production is a reflex secretion (42). Previous studies have demonstrated that milkability traits can be assessed using udder and teat morphological traits of camels, contributing to the selection of high-producing camels (43). Camel teats have a double ductal anatomy with a promising prophylactic effect against mastitis-causing pathogens (44). Although whole-genome sequencing of dromedaries and Bactrian camels has been completed (45, 46), gene annotation remains incomplete. It is necessary to obtain more data for the further annotation of camelids. To the best of our knowledge, the present study is the first RNA-sequencing study of mammary gland developmental gene expression in young and adult camels. Although the sample sizes in the current study were relatively limited, significant differences were found between the morphology and transcription profiles of mammary gland tissue of young and adult camels. Additionally, the three mammary gland samples in each group showed a high correlation, proving that the biological samples within each group had good repeatability.

The morphology of mammary glands undergoes significant changes during development. A distinctive feature of the mammary gland development is its extensive proliferative and differentiation potential, particularly during puberty and pregnancy. Adolescence is the ideal stage for studying mammary gland development. The mammary gland develops its adult form through a biological process referred to as branching morphogenesis (47). Our observations of the mammary gland are consistent with those of previous studies (44), showing that age and lactation strongly influence the microscopic anatomy of the camel mammary gland.

Extensive experimental efforts have been made to investigate the endocrine signaling and pathways that control the proliferation and differentiation of mammary epithelial cells. Such studies have reported the regulatory roles of multiple signaling pathways in mammary gland proliferation and development. Several studies have revealed that the Wnt pathway is required at the earliest stage of mammary development in the embryo for specification of the mammary placode and initiation of mammary morphogenesis (48) and in postnatal development for proper stem cell maintenance, branching morphogenesis, and mammary alveolar development (49, 50). The Hedgehog signaling pathway is also necessary for mammary gland development and distinguishes the mammary gland from other epidermal appendages (51, 52). Mammary gland development involves complex interactions among various hormones. The mammary gland is composed of a branched ductal system consisting of milk-producing epithelial cells that form ductile tubules surrounded by a layer of myoepithelial cells that contract in response to oxytocin stimulation, thereby releasing milk (53). After the embryonic and prepubertal stages, further development of the mammary glands is highly dependent on hormones such as steroids, which control the development of breast ducts and alveoli (53). The results of RNA sequencing and histomorphological identification of the mammary glands of young and adult camels lend further support to these pathways and hormones regulating mammary gland development in animals.

The functions of genes involved in these pathways require further investigation. Comprehensive PPI network analysis identified ten hub genes. Among these, variations in the *BTRC* gene have been reported to influence spermatogenesis and mammary gland development in mice (40). *PRKAB2* and *PRKAG3* have been implicated in animal growth, slaughter, and meat quality regulation (38). In pigs, *PLCB4* is a candidate gene for growth, development, and meat production (39). Among these non-core genes, *GLI1* regulates the glioma-associated oncogene homolog 1 (*GLI1*) activator and causes mammary bud formation failure in mice (54). The *WIF1* gene is expressed at high levels in normal human breast cells and mammary tissues and maintains mammary gland development (55). *DKK2* and *FZD3* are closely related to the Wnt signaling pathway during embryonic mammary gland development (56). The Wnt ligand *WNT4* plays a critical role in normal mammary gland development. Our study identified a set of crucial genes that might exert their functions by regulating the Wnt, Hedgehog, and sex hormone signaling pathways. However, additional functional experiments are required to confirm this hypothesis.

In summary, this study is of great value for identifying critical genes involved in mammary gland development and can inform subsequent studies on the progression mechanism of mammary gland development in camels. However, there were some limitations. The present study is limited by the small samples size of each group. Three camels were used as replicates at each stage. The findings of the study need to be verified in the future through studies with an expanded sample size. Additionally, there are differences in genetic information between individual animals, which may affect the results of the differential expression analysis of the mammary glands (57). Although distinct differences in the function and structure of mammary glands have been found between young and adult camels, mammary gland growth and development are complex biological processes, and inclusion of more sampling and sequencing time points might help to elucidate the molecular change mechanisms of the underlying biological processes and better reflect the continuous characteristics of dynamic gene changes during mammary gland development.

## 5. Conclusion

The Bactrian camel is a versatile animal with high ecological and economic value in remote desert areas. Milk synthesis is an essential function of the mammary glands in mammals. This study described the microstructure and transcriptome profiles of the mammary gland parenchyma of young and adult camels. Differential expression analysis revealed 2,851 DEGs, of which 1,420 were upregulated and 1,431 were downregulated. Functional enrichment analysis revealed significant enrichment of the Wnt, Hedgehog, oxytocin, and insulin signaling pathways and steroid biosynthesis. The *PRKAB2*, *PRKAG3*, *PLCB4*, *BTRC*, *GLI1*, *WIF1*, *DKK2*, *FZD3*, and *WNT4* genes involved in these pathways should be considered as important candidate genes for mammary gland development. Nevertheless, the potential functions of these genes still require further verification. This study revealed DEGs and signaling pathways related to mammary gland development in Bactrian camels and laid a theoretical foundation for the improvement of milk production traits in camels.

## Data availability statement

The datasets presented in this study can be found in online repositories. The names of the repository/repositories and accession number(s) can be found below: https://www.ncbi.nlm.nih.gov/bioproject; PRJNA946168.

## Ethics statement

The animal study was reviewed and approved by Ethics Committee of Xinjiang University. Written informed consent was obtained from the owners for the participation of their animals in this study.

## Author contributions

JY conceived the study. H Yao writing-original draft and writing-review and editing. H Yao, ZD, and XL conducted conceptualization and writing-review and editing. ZZ, WM, ZH, H Yan, YW, ZH, ZW, and GC collected the experimental samples. All authors contributed to the article and approved the submitted version.

## Funding

This work was financially supported by the Key Technology Research and Development Program in Xinjiang Uygur Autonomous Region (2018B01003), the National Key Research and Development Projects of China (2019YFC1606103), and the Postgraduate Scientific Research Innovation Program of Xinjiang Uygur Autonomous Region (XJ2019G026).

## Conflict of interest

GC was employed by Xinjiang Wangyuan Camel Milk Limited Company.

The remaining authors declare that the research was conducted in the absence of any commercial or financial relationships that could be construed as a potential conflict of interest.

## Publisher’s note

All claims expressed in this article are solely those of the authors and do not necessarily represent those of their affiliated organizations, or those of the publisher, the editors and the reviewers. Any product that may be evaluated in this article, or claim that may be made by its manufacturer, is not guaranteed or endorsed by the publisher.

## Footnotes

^1^ https://magic.novogene.com/customer/main#/loginNew

^2^ http://string-db.org/
